# MAPK4 predicts poor prognosis and facilitates the proliferation and migration of glioma through the AKT/mTOR pathway

**DOI:** 10.1002/cam4.5859

**Published:** 2023-03-31

**Authors:** Jing Ren, Shijun Zheng, Lei Zhang, Jia Liu, Haowei Cao, Sicheng Wu, Yixin Xu, Jinmin Sun

**Affiliations:** ^1^ Jiangsu Key Laboratory of Brain Disease Bioinformation, Research Center for Biochemistry and Molecular Biology Xuzhou Medical University Xuzhou Jiangsu China; ^2^ Department of Infectious Diseases, Qingdao Jimo People's Hospital Qingdao Shandong China; ^3^ Department of Neurosurgery The Affiliated Hospital of Xuzhou Medical University Xuzhou Jiangsu China; ^4^ Department of Pathology The Affiliated Hospital of Xuzhou Medical University Xuzhou Jiangsu China; ^5^ Department of General Surgery The Affiliated Hospital of Xuzhou Medical University Xuzhou Jiangsu China; ^6^ Institute of Digestive Diseases Xuzhou Medical University Xuzhou Jiangsu China; ^7^ Laboratory of Clinical and Experimental Pathology, Department of Pathology Xuzhou Medical University Xuzhou Jiangsu China

**Keywords:** AKT/mTOR, glioma, MAPK4, migration, proliferation

## Abstract

**Background:**

Mitogen‐activated protein kinase 4 (MAPK4) is an atypical member of the mitogen‐activated protein kinase (MAPK) family. We report here that MAPK4 is overexpressed in glioma. The clinical significance, biological roles and underlying molecular mechanisms through which MAPK4 acts in glioma remain unclear.

**Methods:**

Analysis of MAPK4 expression and associated survival in glioma patients was performed based on data obtained from The Cancer Genome Atlas (TCGA) and Chinese Glioma Genome Atlas (CGGA) databases and confirmed in human glioma tissue by immunohistochemistry. MAPK4 function and pathway enrichment were analyzed through Gene Set Enrichment Analysis (GSEA) and Gene Ontology (GO). The viability and migration ability of MAPK4‐silenced glioblastoma multiforme (GBM) cells were evaluated using CCK8 and transwell assays, respectively, and cell cycle and apoptosis analyses were performed using flow cytometry. Immunoblotting was used to analyze the protein level in MAPK4 knockdown glioma cells. We also analyzed the correlation of MAPK4 expression with immune infiltration and immune checkpoints in glioma.

**Results:**

MAPK4 was overexpressed in IDH wild‐type (wt) and 1p/19q non‐codeletion gliomas. MAPK4 expression predicted poor prognosis of glioma patients. MAPK4 was significantly related to functional states, including stemness, metastasis, cell cycle, differentiation and proliferation, in glioma at single‐cell resolution. MAPK4 silencing inhibited proliferation and migration and induced G1 cell cycle arrest in glioma cells via the AKT/mTOR pathway. In vivo, MAPK4 knockdown markedly suppressed the growth of primary glioma. In addition, MAPK4 expression correlated negatively with the infiltration of plasmacytoid DC cells, CD8^+^ T cells and T helper cells. Moreover, MAPK4 expression correlated positively with expression of the main immunoinhibitor checkpoint molecules and some chemokines in glioma.

**Conclusion:**

MAPK4 functions as a prognostic indicator in glioma and promotes the proliferation and migration of GBM cells through the AKT/mTOR pathway. MAPK4 may participate in immune infiltration and the expression of immune checkpoints in the glioma microenvironment.

## Graphical abstract

MAPK4 promotes the proliferation and migration of GBM cells by AKT/mTOR pathway. Aberrantly overexpressed MAPK4 in GBM activates AKT(T308) and the downstream molecules of mTORC2, AKT(S473) and PKC. Thephosphorylation of AKT and PKC promotes the proliferation and migration of GBMcells. MAPK4 promotes the proliferation and migration of GBM cells through amechanism involving AKT activation.

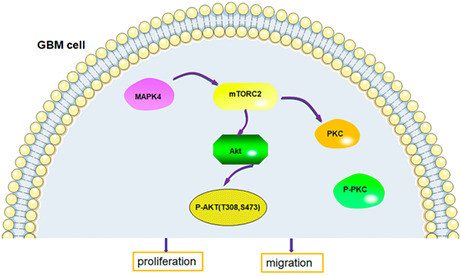

## 1 BACKGROUND

Despite standard‐of‐care treatment comprising maximal surgical resection followed by radiation and chemotherapy, median survival rates for glioma patients remain stubbornly low.^1^ The 2021 WHO classification of tumors of the central nervous system, 5th edition (WHO CNS 5), describes molecular biomarkers that can be used to distinguish subtypes of glioma,^2^ giving additional benefits and meaningful instructions to the clinic. Clinical trials in which the expression of many classic targets, such as the P53, retinoblastoma and epidermal growth factor receptor gene, failed due to the involvement of a complex regulatory network.^3^ More effective molecular targets are urgently needed. Immunotherapy, such as anti‐PD‐1/anti‐PD‐L1, has dramatically improved outcomes in patients some types of tumors. However, in most glioma cancer patients, durable clinical responses to immunotherapy are not achieved due to the tumor‐intrinsic resistant phenotypes and immunosuppressive microenvironment.^4^ Currently, we still lack effective therapeutic targets that can be used to overcome tumor‐intrinsic resistant phenotypes and immunosuppressive microenvironments in glioma. In WHO CNS 5, “MAPK altered” is used in the classification of molecular types of glioma. The expression of several genes associated with the MAPK pathway, including those encoding fibroblast growth factor receptor 1 and v‐raf murine sarcoma viral oncogene homolog B1 (BRAF) p. V600E, is commonly altered in glioma; genes whose expression is less frequently altered include the genes that encode neurotrophin receptor kinase 1/2/3 (NTRK1/2/3), mesenchymal‐epithelial transition factor (MET) and mitogen‐activated protein kinase kinase 1 (MAP2K1), and increased expression of these genes can also lead to MAPK pathway activation. MAPK4 is as an atypical member of the MAPK family that is involved in multiple physiological processes.^5^, ^6^, ^7^ MAPK4 lacks the canonical Thr‐X‐Tyr activation motifs that mediate phosphorylation activation by the dual Ser/Thr and Tyr MAPK kinase (MAPKK).^8^, ^9^

In recent years, a promoting role of MAPK4 in a variety of tumors has been reported, although the physiological basis for this promotion remains unclear. MAPK4 promotes the proliferation of colon cancer,^10^ lung cancer,^10^ prostate cancer,^11^ and triple‐negative breast cancer^12^ and reduces tumor sensitivity to inhibitors of ADP‐ribose polymerase in cervical cancer cells^13^ and PI3K in triple‐negative breast cancer.^12^ AKT/mTOR plays a crucial role in proliferation, migration and metabolism. mTOR includes two different complexes, mTORC1 and mTORC2. AKT (S473) is mainly activated by mTORC2, which works together with PDK1 which fully activate AKT.^11^ MAPK4 promotes the proliferation of colon cancer and lung cancer by noncanonical AKT/mTOR,^10^ while MAPK4 facilitates prostate cancer growth and castration resistance by activating the GATA2/AR and AKT pathways in parallel.^11^ However, the role of MAPK4 in AKT activation, as well as the expression, clinical significance, biological roles and underlying molecular mechanisms through which MAPK4 acts in glioma, is still obscure.

Here, we report that elevated expression of MAPK4 correlates with reduced survival time. Aberrant expression of MAPK4 also correlated with poor clinicopathological characteristics and disease progression of glioma. MAPK4 may be a prognostic indicator and diagnostic marker. MAPK4 was found to promote the proliferation and migration of glioma cells via the AKT/mTOR pathway by bioinformatic analyses and experimental verification. In addition, MAPK4 may participate in the regulation of immune infiltration and main immune‐inhibitor checkpoint molecules.

## 2 MATERIALS AND METHODS

### 2.1 Clinical glioma specimens

Clinical glioma specimens of patients (*n* = 97) with surgical resection were selected from the Affiliated Hospital of Xuzhou Medical University. The samples were diagnosed by two pathologists according to the criterion of central nervous system tumor in 2016 WHO. This study obtained the approval from Ethics Committee of the Affiliated Hospital of Xuzhou Medical University (Approval number. XYFY2018‐KL056‐01) and conducted in conformity to the Declaration of Helsinki.

### 2.2 Cell culture and transfection of lentivirus

Human normal brain glial cells (HEB) and GBM cells (T98G, U87, U251, LN229 and U118) from American Type Culture Collection were cultured in DMEM supplemented with 10% fetal bovine serum (FBS, Life Technologies).The shRNA lentivirus for MAPK4 was packed by Genechem. Sequences of MAPK4 shRNA were designed according to the reference (10) as follows, MAPK4‐sh1, GGGTTGGTAACAAAGTGGT; MAPK4‐sh2, CGGGAGGAAGACAAGGACG. U87 and T98G cells were seeded and transfected with lentivirus when the cells grew to 20%–30%. Cells transfected with MAPK4 shRNA lentivirus were screened by puromycin.

### 2.3 Data acquisition

RNA sequencing data of glioma was obtained from The Cancer Genome Atlas (TCGA) database (*n* = 703), Chinese Glioma Genome Atlas (CGGA) database (*n* = 1152) and Genotype‐tissue expression (GTEx) database. In CGGA, the incomplete data were deleted and 413 glioma specimens (batch I) and 273 glioma specimens (batch II) were used. Glioma cases with complete clinical data, including pathological type, age, gender, grade, primary or recurrence status, survival time, radiotherapy, chemotherapy, IDH status, 1p/19q status, and MGMT status were selected for the analysis and cases with incomplete clinical data were excluded. The median value of MAPK4 mRNA expression in the datasets was selected for stratifying the patients. Gene Expression Omnibus (GEO) database (GSE131928, GSE102130, GSE57872 and GSE103224) was used. Broad Institute Single‐Cell Portal was used to explore the MAPK4 expression in different sub‐cluster of glioma. To explore the distinct functional states of glioma cells regulated by MAPK4, dataset 1 (accession: GSE102130), dataset 2 (accession: GSE84465) and dataset 3 (accession: GSE103224) were used.

### 2.4 Immunohistochemistry (IHC) analysis

IHC was performed according to standard procedures as previous described.^14^ In brief, glioma tissues embedded in paraffin were sliced into sections (4‐μm). Then, the sections were deparaffinized, rehydrated and blocked with 10% goat serum after antigen retrieval by sodium citrate buffer. The primary antibodies for MAPK4 (Affinity, 1:300), p‐AKT (Thr308) (Affinity, China, 1:100), p‐AKT (Ser473) (Proteintech, 1:250) and horseradish peroxidase labeled secondary antibodies were used followed by hematoxylin staining. Two pathologists cored the immunoreactive score by a double‐blind method. Each specimen was scored for the intensity of the cell plasma and for the positive extent. The intensity was determined as 0 (negative), 1 (weak), 2 (moderate), 3 (strong) and the positive extent was determined as 1 (0%), 2 (1%–24%), 3 (25%–49%), 3 (50%–74%) and 4 (75%–100%). The multiplicative value of the intensity and the positive extent was the final score which was used to perform statistical analysis.

### 2.5 Cell viability evaluation

GBM cells (5000/well) were seeded and the viability was evaluated by CCK8 (Vicmed, VC5001) in 0, 24, 48 and 72 h according to the protocol. Absorption value in A450 was measured by a microplate reader.

### 2.6 Cell cycle and cell apoptosis

Cell cycle kit (Keygen Biotech, China) was used to stain the cells after collecting. In brief, cells were fixed, washed and stained by propidium Iodide (PI). For cell apoptosis, cell apoptosis kit (Fcmacs, FMSAV647) was used following the instruction. 5 μL Annexin V‐Alexa Fluor 647 and 10 μL PI in 100 μL 1× binding buffer were incubated with cells after collecting. All samples were detected by flow cytometry immediately (FACS Canto II).

### 2.7 Transwell assay

Upper chambers containing DMEM with free of FBS were placed inside the lower chambers containing medium with 10% FBS. Cells (1 × 10^5^) were starved and seeded in the upper chanmper in the incubator. The chambers were taken out and fixed with 4% paraformaldehyde. The migrated cells were stained with 0.1% crystal violet and cells numbers in five randomly view fields were calculated and analyzed statistically.

### 2.8 Intracranial GBM xenograft model

U87 cells (5 × 10^5^) transfected with MAPK4‐shRNA lentivirus and luciferase lentivirus and the control cells were stereotactically transplanted into the right cerebral cortex of female nude mice (5 weeks). The position of the microsyringe needle was 2 mm to the right of bregma, 1 mm to the front of coronal suture and 3 mm below the surface of the skull via a stereotactic instrument. The drill holes were blocked with bone wax and the incisions were sutured. Tumor volume was recorded in vivo using Night OWL II LB 983 in vivo imaging system bioluminescent Imaging (Berthold Technologies) D‐Luciferin potassium salt(VICMED) was used.

### 2.9 Survival analysis

Survival analysis was performed by Kaplan–Meier method and Cox regression. R packages “survival” and “survminer” were used to analyze and plot the survival curves.

### 2.10 Bioinformatics analysis

For the function and pathway enrichment, co‐expression genes of MAPK4 were analyzed in TCGA glioma database by a Pearson correlation coefficients (|*r*| >0.4 and *p* < 0.001).The package R “clusterProfiler” was used to perform GO analysis of co‐expressed genes of MAPK4 from TCGA database. GO circle plot and GO Chord were analyzed and plotted by R package GOplot (1.0.2) and ggplot2 (3.3.3). GAEA was performed by cluster Profler R package (v.3.6.3). To analyze the correlation between MAPK4 and immunity, web server TIMER was used to compare the tumor infiltration levels in GBM with different somatic copy number alterations in MAPK4. The correlation between checkpoint molecules and MAPK4 expression were also analyzed by TIMER. The abundance of immune cell types and correlation between immune checkpoints and MAPK4 expression were analyzed by R package “GSVA” with Single‐Sample GSEA (ssGSEA) algorithms.

### 2.11 Western blot analysis

Protein from GBM cells was collected, separated by 10% SDS/PAGE and electrotransferred to a nitrocellulose (NC) membrane. The primary antibodies anti‐MAPK4 (1:1000, Affinity), anti‐p‐AKT (Thr308) (Affinity, 1:1000), anti‐p‐AKT (Ser473) (Proteintech, 1:1000), anti‐AKT (Proteintech, 1:1000), anti‐PKC (Proteintech, 1:1000), and anti‐p‐PKC (Proteintech, 1:1000) and a horseradish peroxidase (HRP)‐labeled secondary antibody (1:10000) were used. Chemiluminescent HRP substrate was used for band detection.

### 2.12 Statistical analyses

The chi‐square (*χ* ^*2*^) test, Pearson correlation and Spearman correlation analysis were used to evaluate correlations. The survival analysis was performed using the Kaplan–Meier method. Univariate and multivariate Cox analyses were conducted using the R package “survival”. Student's *t*‐test was used to determine the statistical significance of differences between two groups, and differences among more than two groups were analyzed by one‐way ANOVA and the Kruskal–Wallis test.

## 3 RESULTS

### 3.1 Aberrant overexpression of MAPK4 in glioma correlates strongly with poor clinicopathological characteristics

**FIGURE 1:**
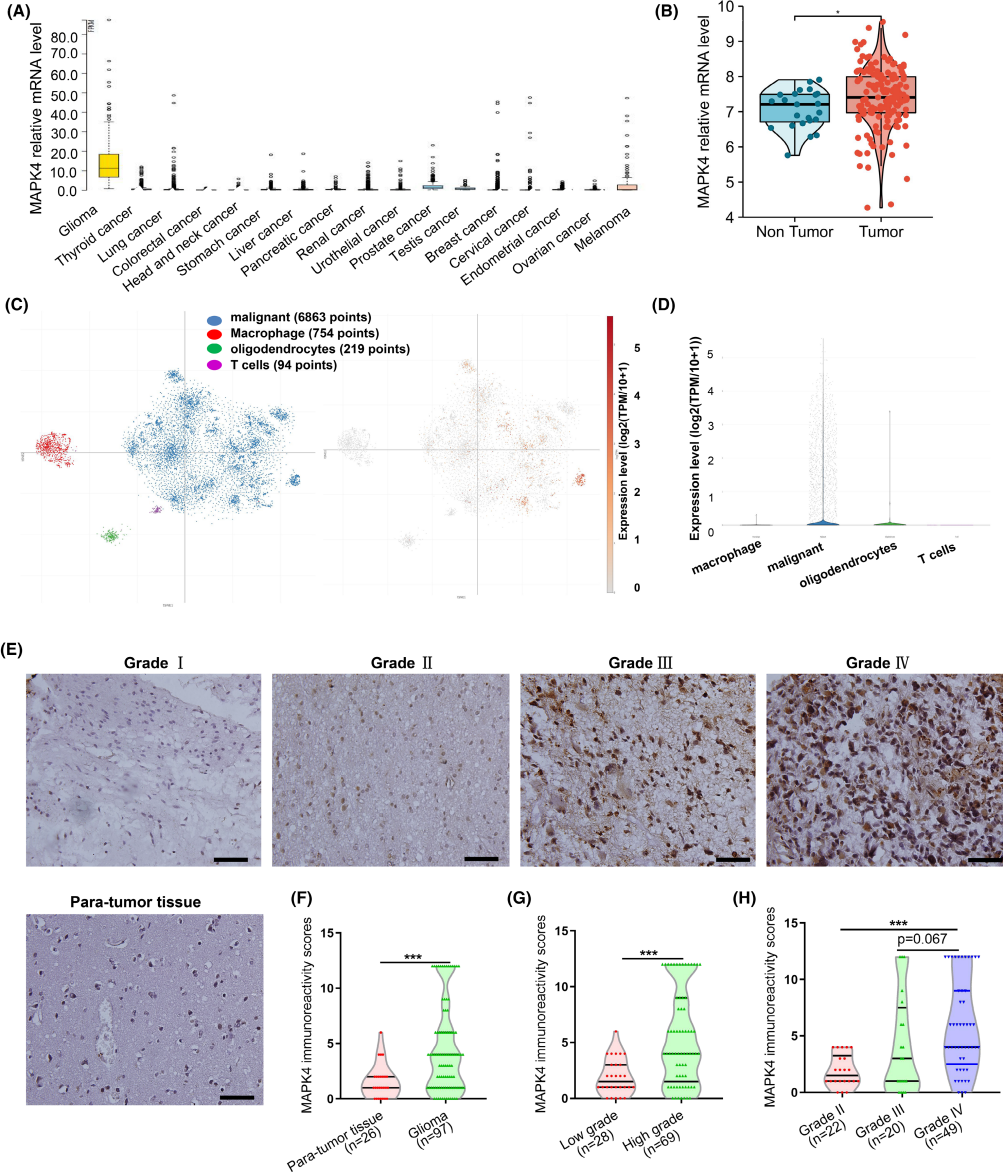
Aberrantly overexpressed MAPK4 is significantly correlated with the poor clinicopathological characters of glioma. (A) MAPK4 mRNA distribution in pan‐cancer. (B) The level of MAPK4 mRNA was obviously elevated in glioma compared with non‐tumor tissue. (C, D) MAPK4 expression in different sub‐cluster of glioma from single‐cell RNA‐sequencing data. (E) Representative images of MAPK4 IHC staining in glioma tissue and para‐tumor tissue. Scale bars, 50 μm. (F) MAPK4 protein level was increased in glioma tissue (*n* = 97) relative to para‐tumor tissue (*n* = 26). (G, H) The protein level of MAPK4 in different grade glioma *p < 0.05, ***p < 0.001.

As shown by the gene expression profiles of different cancers, MAPK4 was present at high levels in glioma, accompanied by the enriched expression of MAPK4 in diverse cancers (Figure 1A). Enhanced expression level of MAPK4 was found in glioma tissues than in normal tissues (Figure 1B). Single‐cell RNA‐seq data were used to further identify the cell types expressing MAPK4 in GBM. The results indicated that MAPK4 was predominantly expressed in tumor cells, other than immune cells or other non‐tumor cells (Figure 1C,D). To further validate the expression of MAPK4 in specific cell types, we measured MAPK4 expression in human glioma and para‐tumor tissues by IHC. The protein levels of MAPK4 were higher in high‐grade glioma compared with low‐grade glioma (Figure 1E–H).

**FIGURE 2:**
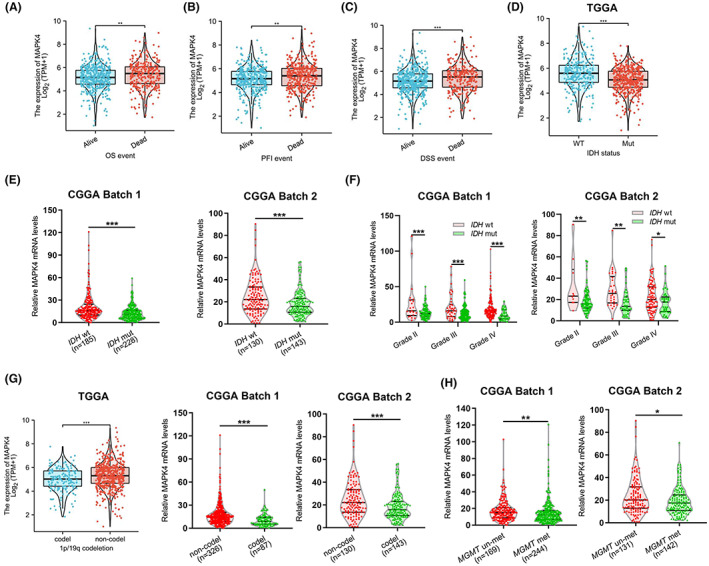
MAPK4 expression in groups with different clinical parameters or molecular markers. (A–C) The expression of MAPK4 in different groups in view of the prognosis of glioma patients, including the OS, PFI and DSS event of glioma patients. (D–F) Higher expression level of MAPK4 in *IDH* wt groups compared with IDH mut groups from TCGA and CGGA. (G) MAPK4 expression was obviously elevated in 1p/19q non‐codeletion group compared with I 1p/19q codeletion group. (H) MAPK4 expression was obviously elevated in MGMT un‐methylation cohort group compared with MGMT methylation cohort group.*p < 0.05, **p < 0.01, ***p < 0.001.

As the clinical significance of MAPK4 expression in gliomas remain unclear, we analyzed the expression of MAPK4 reported in the TCGA database for glioma tissue obtained from patients whose survival parameters differed. The analyzed parameters included overall survival (OS), disease‐specific survival (DSS) and disease‐free interval (DFI). We found that MAPK4 expression was increased in the dead groups compared with the live groups (Figure 2A–C). Molecular markers such as *IDH* genotype, 1p/19q and O6‐methylguanine‐DNA methyltransferase (MGMT) have been widely used in glioma patients and supply valuable clinical information.^15^ We further assessed the role of MAPK4 in different molecular subtypes of glioma using the data obtained from the CGGA and TCGA. The results showed that MAPK4 expression was elevated in the *IDH* wt (Figure 2D–F), 1p/19q non‐codeletion (Figure 2G) and MGMT un‐methylation cohorts (Figure 2H).

MAPK4 expression in gliomas with different clinicopathological characteristics was analyzed based on the TCGA data. Overexpression of MAPK4 was significantly correlated with *IDH* genotype, 1p/19q codeletion and age (Table 1). Correlation analysis of MAPK4 IHC staining with clinicopathologic parameters was performed, and increased protein levels of MAPK4 were found to be significantly correlated with the WHO grade of glioma (Table 2). Aberrant MAPK4 expression was significantly associated with poor clinicopathological characteristics of glioma. These data implicate MAPK4 in glioma progression and suggest that it may play a promoting role in glioma.

**TABLE 1:** Association between MAPK4 mRNA expression and the clinical parameters of glioma patients in TCGA.

Characteristic	Low expression of MAPK4	High expression of MAPK4	*p*
*n*	348	348	
WHO grade, *n* (%)			0.718
G2	116 (18.3%)	108 (17%)	
G3	118 (18.6%)	125 (19.7%)	
G4	81 (12.8%)	87 (13.7%)	
IDH status, *n* (%)			<0.001
WT	91 (13.3%)	155 (22.6%)	
Mut	252 (36.7%)	188 (27.4%)	
1p/19q codeletion, *n* (%)			0.003
Codel	103 (14.9%)	68 (9.9%)	
Non‐codel	243 (35.3%)	275 (39.9%)	
Age, meidan (IQR)	42 (34, 57)	48 (36, 60)	0.006

Abbreviations: IDH, Socitrate Dehydrogenase; IQR, Interquartile Range; WHO, World Health Organization.

**TABLE 2:** MAPK4 IHC staining and clinicopathological characteristics of 96 glioma patients.

Variable	Number (*n*)	MAPK4 staining			
		Low (%)	High (%)	*Χ* ^*2*^	*p*
Sex				0.901	0.342
Male	61	43 (70.5)	18 (29.5)		
Female	36	22 (61.1)	14 (38.9)		
Age				0.139	0.709
<50 years	42	29 (69)	13 (31)		
≥50 years	55	36 (65.5)	19 (34.5)		
Tumor size				0.042	0.837
<5 cm	30	22 (73.3)	8 (26.9)		
≥5 cm	31	22 (71.0)	9 (29.0)		
WHO Grade				15.410	0.000
Low (I‐II)	28	27 (96.4)	1 (3.6)		
High (III‐IV)	69	38 (55.1)	31 (44.9)		

### 3.2 Increased MAPK4 expression may be a prognostic indicator and is significantly correlated with glioma progression

**FIGURE 3:**
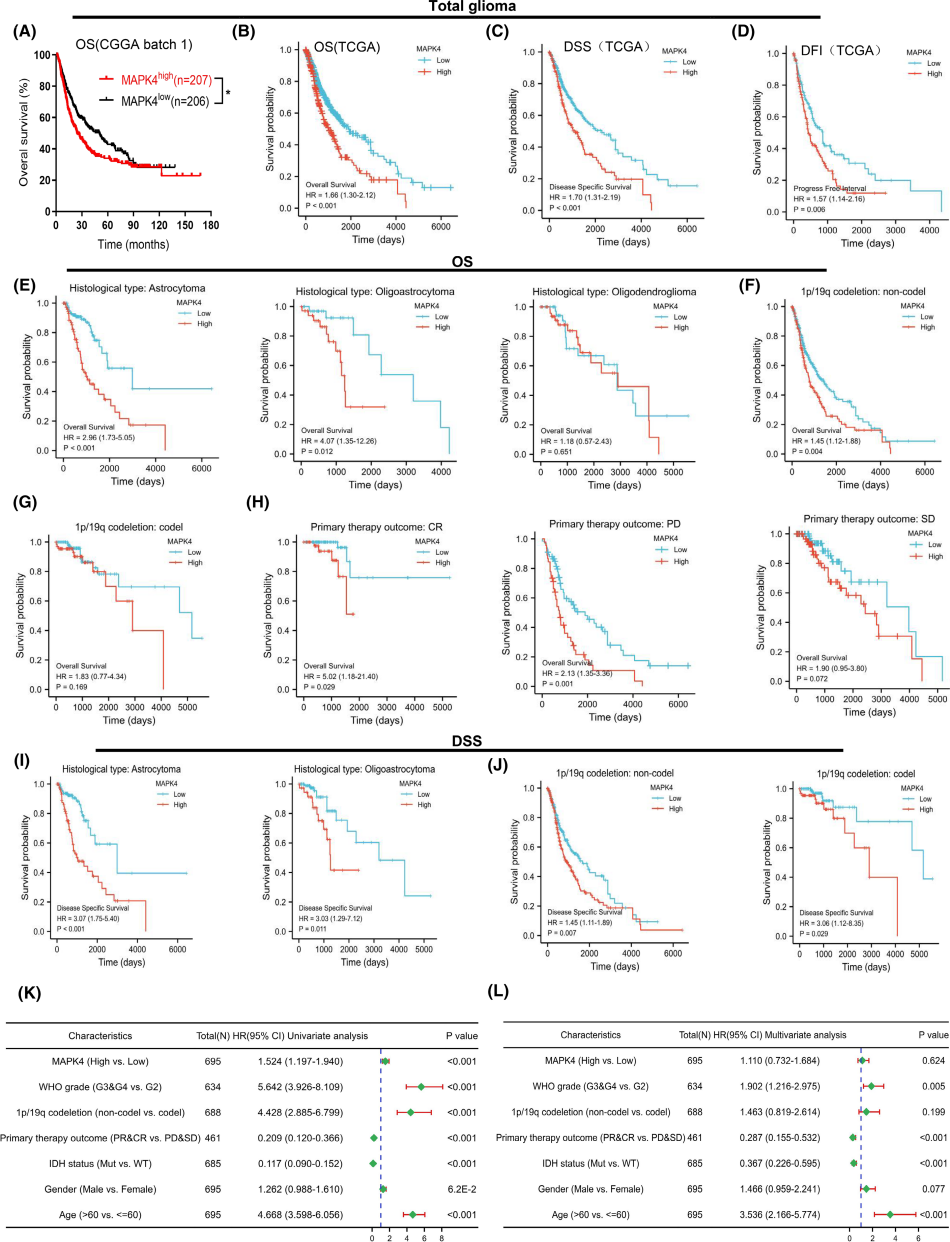
Increased MAPK4 expression may be a prognostic indicator and is significantly correlated with glioma progression. Overexpressed MAPK4 was correlated with poor prognosis of total glioma patients in OS (A, B), DSS (C) and DFI (D). (E) Overall survival analysis of MAPK4 expression of different histological types. (F, G) Overall survival analysis of MAPK4 in 1p/19q non‐codeletion and 1p/19q codeletion group. (H) Overall survival analysis of MAPK4 in deferent primary therapy outcome groups. (I, J) Disease specific survival analysis of MAPK4 in astrocytoma, oligoastrocytoma, non‐codel 1p/19q codeletion and codel 1p/19q codeletion groups. (K, L) The univariate (K) and multivariate (L) cox regression analysis were performed in TCGA cohort and shown by forest plot.

In view of the increased expression of MAPK4 observed in glioma, we evaluated the prognostic value of elevated MAPK4 expression in glioma based on data from the CGGA and TCGA databases. A high level of MAPK4 expression predicted poor prognosis in glioma patients as reflected by OS (Figure 3A,B), DSS and PFI (Figure 3C,D). Analysis of OS in patients grouped by histological type, 1p/19q status and primary therapy outcome, also showed that poor prognosis was accompanied by increased MAPK4 levels in the astrocytoma and oligoastrocytoma, 1p/19q noncodeletion, progressive disease (PD) and complete response (CR) groups (Figure 3E‐H). In addition, the results of DSS analysis in the astrocytoma, oligoastrocytoma and 1p/19q noncodeletion groups were consistent with the OS analyses (Figure 3I,J).

We conducted univariate and multivariate Cox regression analyses to determine the prognostic significance of elevated MAPK4 expression with respect to risk signature and clinicopathologic characteristics. In univariate Cox regression analysis, MAPK4 expression, WHO grade, 1p/19q codeletion and age correlated with overall survival in glioma patients (Figure 3K), whereas in multivariate Cox regression analysis, WHO grade, 1p/19q codeletion and age correlated with overall survival (Figure 3L). These results indicate that MAPK4 upregulation signifies poor prognosis in glioma patients.

### 3.3 Functional states related to MAPK4 expression in glioma at single‐cell resolution

**FIGURE 4:**
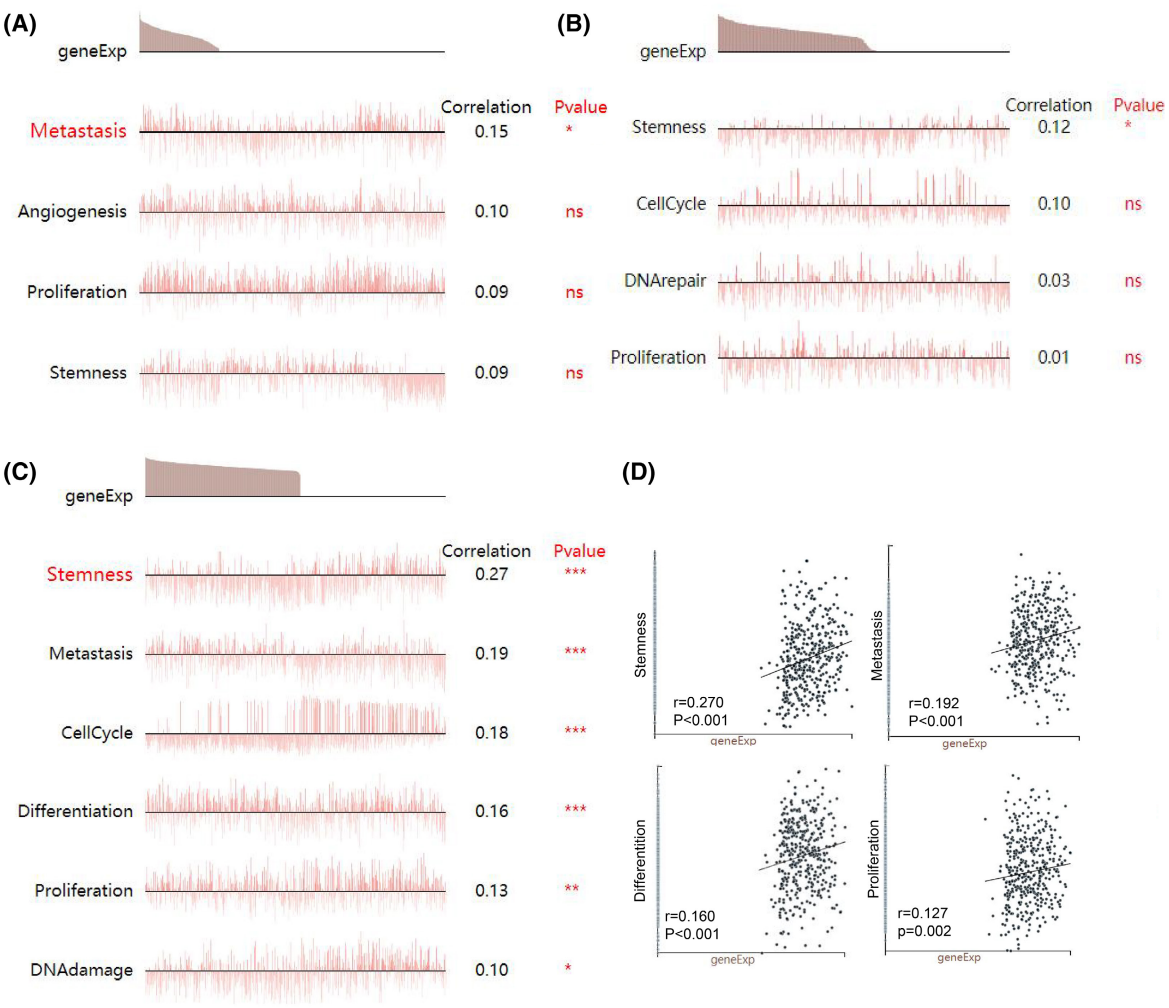
Functional states related to MAPK4 in glioma at single‐cell resolution. The functional states related to MAPK4 in glioma from single cell RNA‐seq dataset 1 (A), dataset 2 (B) and dataset 3 (C, D). *p* > 0.05, *p < 0.05, **p < 0.01, ***p < 0.001.

To determine the functional states of MAPK4 in glioma cells at single‐cell resolution, we queried the highly related functional states of MAPK4 using the Cancer Single‐Cell State Atlas (CancerSEA) web server. As shown in Figure 4A, metastatic functional state was significantly related to MAPK4 expression in the single‐cell RNA‐seq dataset 1 of glioma. Functional states, including inflammation, angiogenesis, proliferation and differentiation, were significantly related to MAPK4 expression in the single‐cell RNA‐seq dataset 2 of glioma (Figure 4B). MAPK4 expression was also significantly related to functional states, including stemness, metastasis, differentiation, proliferation and DNA damage, based on the information reported in single‐cell RNA‐seq dataset 3 of glioma (Figure 4C,D). Collectively, these results suggest that MAPK4 may be involved in functional states, including proliferation, metastasis, angiogenesis and differentiation in glioma.

### 3.4 Knockdown of MAPK4 inhibited proliferation and cell migration and induced cell cycle arrest of GBM cells

**FIGURE 5:**
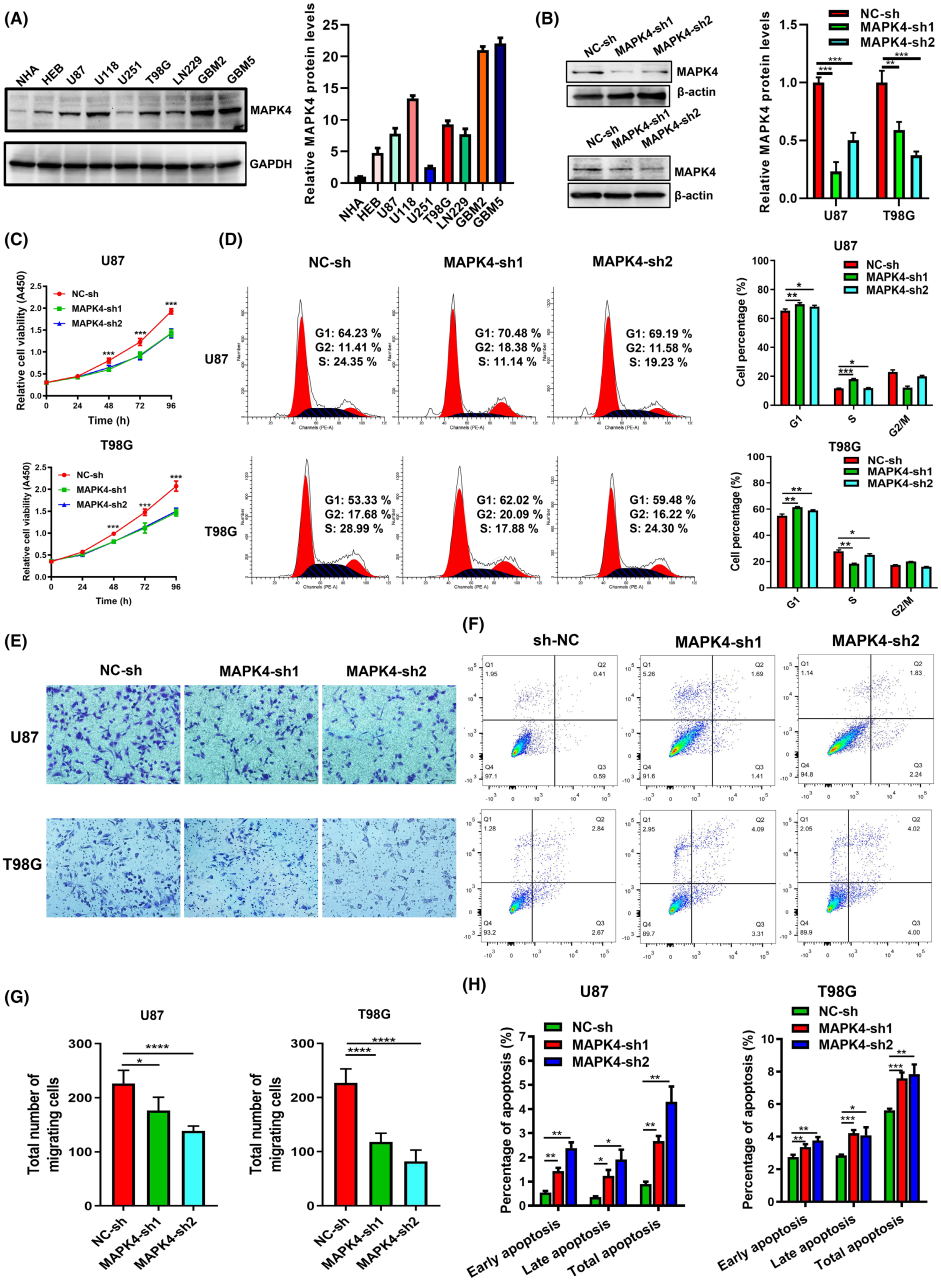
Knockdown of MAPK4 inhibited proliferation and cell migration and induced cell cycle arrest of GBM cells. (A) Immunoblot was used to evaluate the expression of MAPK4 in five GBM cell lines and a normal astrocyte cell line (HEB). Protein intensity was analyzed by densitometry and the levels were normalized to GAPDH. (B) MAPK4 was silenced by shRNA in U87 and T98G cells. Knockdown efficiency was evaluated by immunoblot. Protein intensity was analyzed by densitometry and the levels were normalized to actin. (C) The viability of U87 and T98G cells under MAPK4 knockdown was evaluated by CCK8 after cell seeding. (D) Cell cycle of U87 and T98G cells under MAPK4 knockdown was measured by Flow Cytometry. (E and G) Apoptosis of MAPK4 knockdown U87 and T98G was measured by Flow Cytometry. (F and H) The migration ability of MAPK4 knockdown U87 and T98G cells was assessed by transwell assay. Data represent three independent experiments. Error bars, SD. ns. *p* > 0.05, *p < 0.05, **p < 0.01, ***p < 0.001, ***p < 0.0001.

We measured MAPK4 expression in multiple GBM cell lines and normal astrocyte lines and found enhanced expression of MAPK4 in the indicated GBM cell lines (Figure 5A). U87 and T98G cells were selected for use in the following experiments due to their high expression of MAPK4. To explore its biological role in glioma cells, MAPK4 was knocked down using shRNA lentivirus (Figure 5B). MAPK4 silencing significantly inhibited the proliferation of GBM cells in vitro (Figure 5C). Moreover, MAPK4 silencing induced G1 phase cell cycle arrest (Figure 5D) and promoted GBM cell apoptosis (Figure 5E,G). Migration of U87 and T98G cells was also significantly suppressed by MAPK4 silencing (Figure 5F–H). Our results demonstrate that MAPK4 facilitates proliferation and migration in glioma cells.

### 3.5 The growth of primary glioma is suppressed by MAPK4 silencing

**FIGURE 6:**
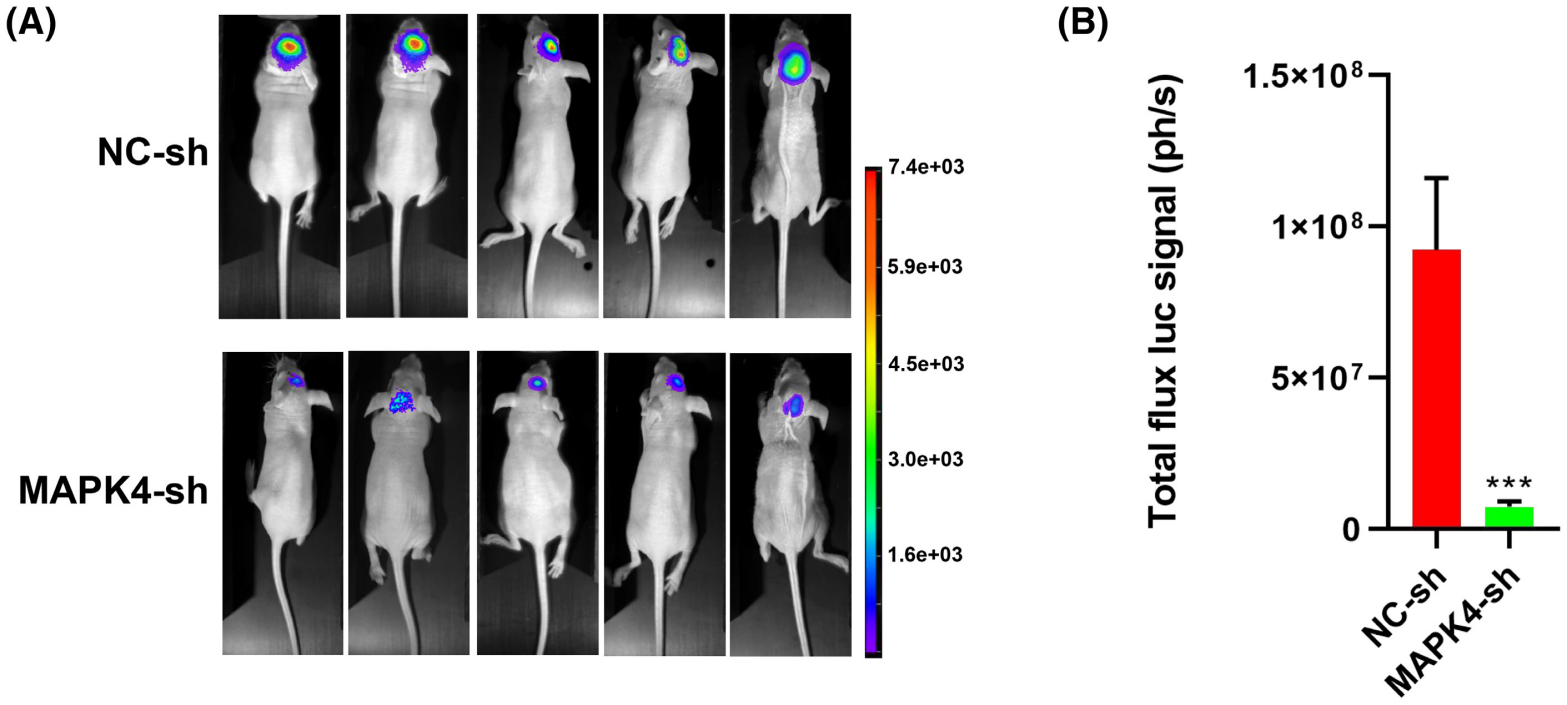
The growth of primary glioma is suppressed by MAPK4 silencing. (A) Luciferase‐labeled MAPK4‐knockdown U87 cells were used to establish the intracranial glioma model and tumor growth was recorded in vivo using bioluminescent imaging. Representative bioluminescent images (A) and the quantification (B) of the U87 tumor‐bearing mice on days 21. The data are shown as mean ± SD (*n* = 5). ***p < 0.001.

To investigate the effect of MAPK4 on the growth of intracranial primary glioma in vivo, we established an intracranial glioma model by using luciferase‐labeled MAPK4‐knockdown U87 cells. As shown in Figure 6, the growth of orthotopic glioma was suppressed by MAPK4 silencing in this model.

### 3.6 Function and pathway enrichment analysis of MAPK4 in glioma

**FIGURE 7:**
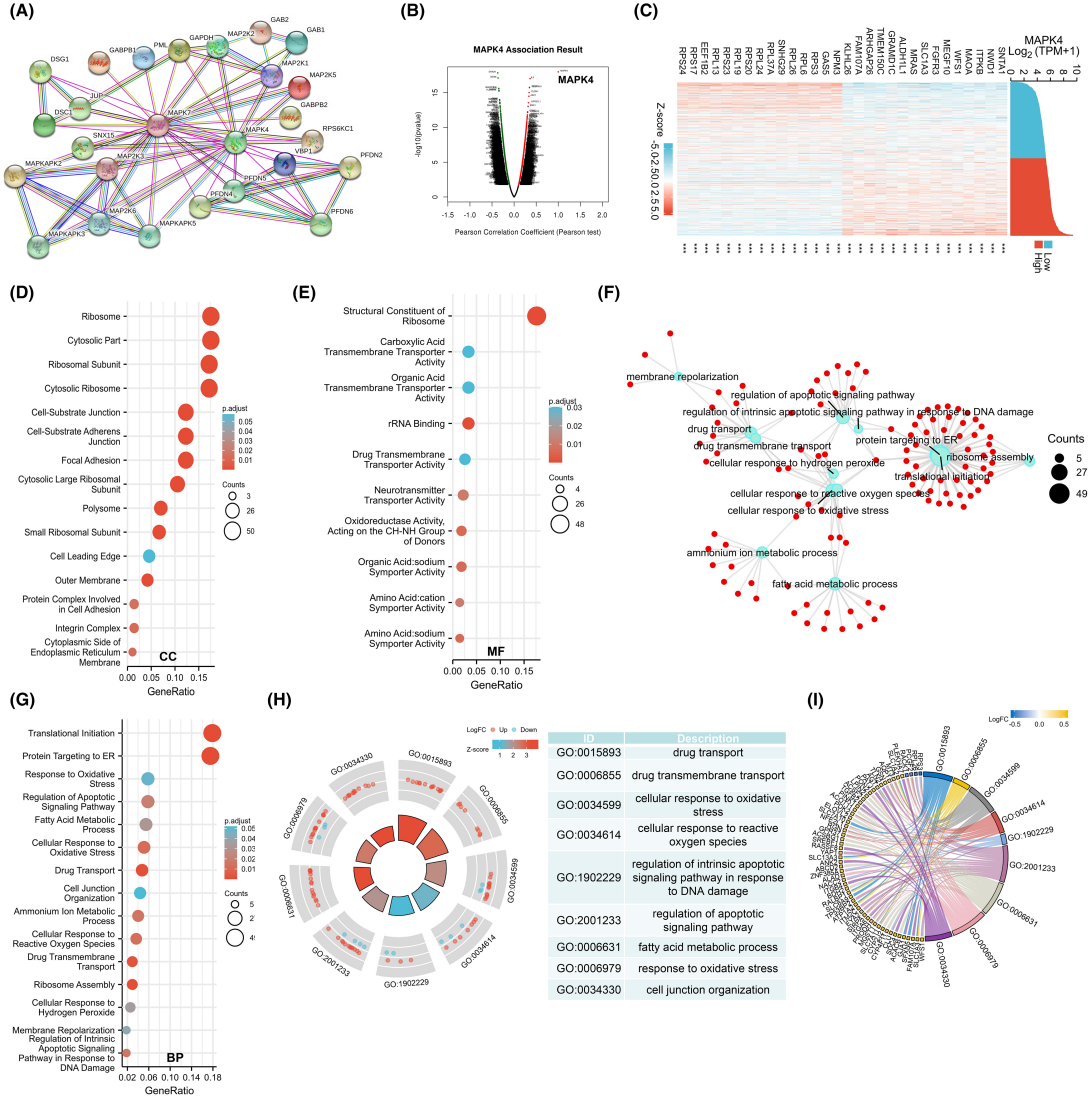
Function and pathway enrichment analysis of MAPK4 in glioma. (A) The MAPK4 network of protein–protein was generated by STRING tool. (B) Volcano Plot showed the genes which associated with MAPK4. (C) Top 15 genes which positively and negatively associated with MAPK4 in glioma. (D) GO enrichment analysis for cellular component of MAPK4 and its co‐expression genes. (E) The enriched molecular function GO terms of MAPK4 and its co‐expression genes. (F) The enriched biological processes molecular function GO terms of MAPK4 and its co‐expression genes. The blue node represents the GO term, the red node represents the molecule, and the line represents the relationship between the GO term and the molecule. (G) GO enrichment analysis of biological processes for MAPK4 and its co‐expression genes. (H) GO circle plot in BP. The bar plots are shown in the inner ring which the height of the bar indicates the significance of the term, and color corresponds to the z‐score. Expression levels (logFC) for the genes in each term are shown in the scatter plots of outer ring. (I) GO Chord plot in BP; the genes are linked via ribbons to their assigned terms. Error bars, SD. ***p < 0.001.

Next, we attempted to identify the functional and physical interaction partners of MAPK4. The protein–protein interaction network in which MAPK4 participates was analyzed by STRING and is shown in Figure 7A. A volcano plot developed using the LinkedOmics web server showed genes associated with MAPK4 (Figure 7B). Heatmaps were used to display the top 15 genes that are positively and negatively associated with MAPK4 in glioma based on information in the TCGA dataset (Figure 7C). We performed GO enrichment and GSEA to identify the biological function of MAPK4 in glioma. The genes coexpressed with MAPK4 were enriched in cellular components (CCs), including focal adhesion, ribosomal subunit, cell‐substrate adheres junction, cell‐substrate junction and integrin complex (Figure 7D). Most of the enriched CC terms were related to cell adhesion, a process that is involved in the proliferation and migration of cancer cells. Molecular functions (MF) and biological processes (BP), such as cell junction organization, neurotransmitter transporter activity, drug transmembrane transporter activity, and drug transport, were also enriched among the MAPK4 coexpressed genes (Figure 7E–I). These results suggest that MAPK4 may play a role in cell junction and drug transport, processes that are involved in multidrug resistance in cancer cells. The relationship between GO terms and specific molecules is shown in the GO enrichment network (Figure 7G). The GO circle plot demonstrated that BP, including drug transport, drug transmembrane transport, cell junction organization, fatty acid metabolic process and response to oxidative stress, may be positively regulated by MAPK4. Most of the molecules associated with those BP terms were upregulated (Figure 7H). In the GO chord plot (Figure 7I), the genes are linked via ribbons to their assigned BP terms. Blue‐to‐red coding next to the selected genes indicates the logFC of expression.

**FIGURE 8:**
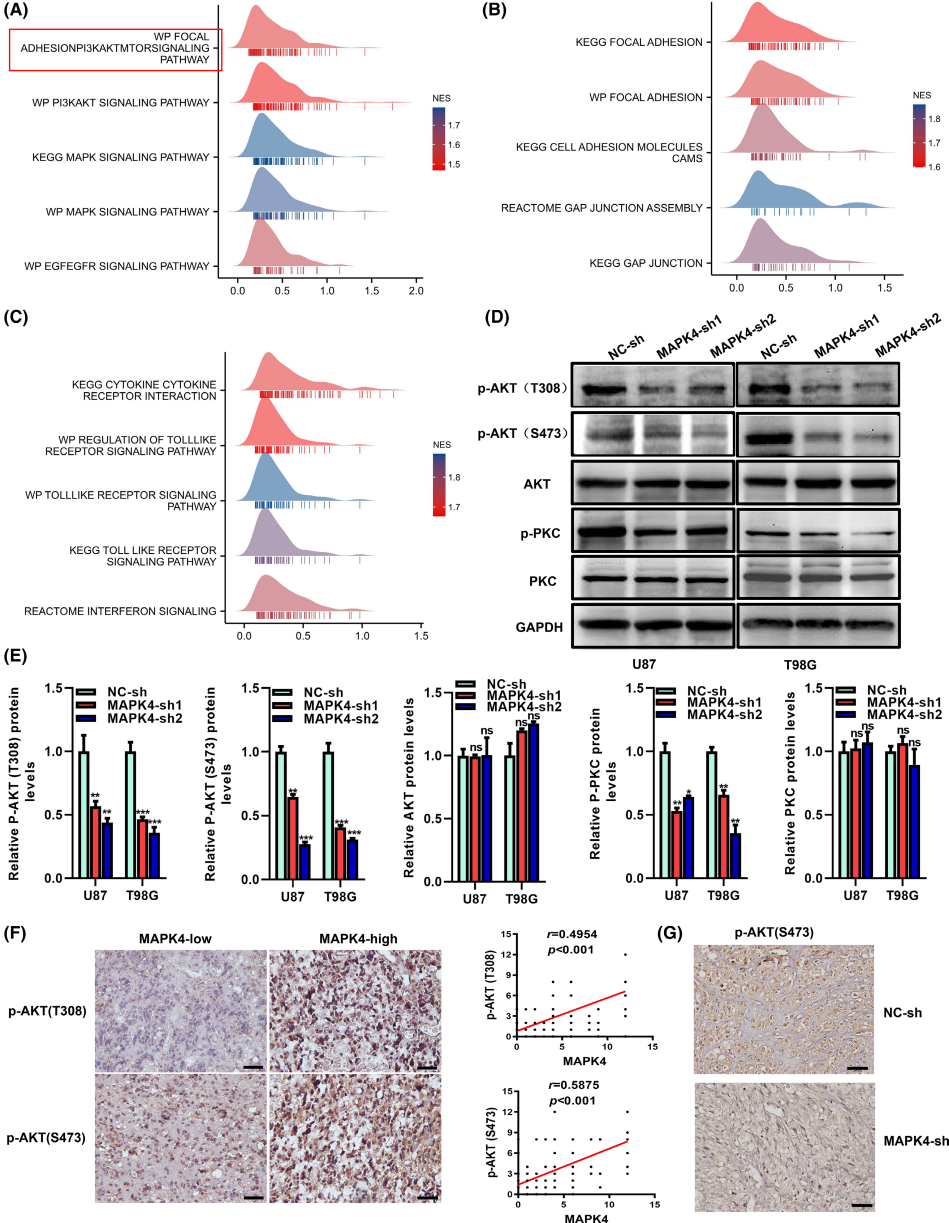
MAPK4 may promote proliferation and migration via the AKT/mTOR pathway. (A–C) GSEA enrichment analysis of MAPK4 and its co‐expression genes in glioma by TCGA data was performed. (D) Immunoblot analyzed the protein level of p‐AKT (T308), p‐AKT (S473), p‐PKC, PKC and AKT in MAPK4 knockdown glioma cells. (E) Protein intensity was analyzed by densitometry and the levels were normalized to GAPDH. (F) The p‐AKT (T308) and p‐AKT (S473) level in human glioma tissues were detected by IHC. The correlation between the level of p‐AKT and MAPK4 were analyzed.(G) The p‐AKT (T308) and p‐AKT (S473) level in mouse glioma tissue from intracranial glioma model were detected by IHC. **p < 0.01, ***p < 0.001.

GSEA also showed that signaling pathways (MAPK, PI3K, Focal_adhesion‐PI3K/AKT/mTOR and EGFR) were significantly enriched in gliomas with high MAPK4 expression (Figure 8A). GSEA was performed using three pathway databases (KEGG, WikiPathways [WP] and Reactome). Pathways related to tumor cell adhesion, such as focal adhesion and gap junction, were enriched, consistent with the results of the GO enrichment analysis (Figure 8B). Moreover, immune‐related signaling pathways, such as the Toll‐like receptor signaling pathway, interferon signaling cytokine and cytokine–cytokine receptor interaction were significantly enriched (Figure 8C).

### 3.7 MAPK4 may promote proliferation and migration via the AKT/mTOR pathway

Signaling pathways, including the MAPK, PI3K, focal adhesion‐PI3K/AKT/mTOR, and EGFR pathways, were enriched in gliomas with high MAPK4 expression (Figure 8A). According to previous studies, MAPK4 promotes tumor progression by activating the AKT/mTOR pathway in a noncanonical manner.^10^ Therefore, we performed immunoblotting experiments in which we analyzed the protein expression of p‐AKT (T308), p‐AKT (S473) and AKT in MAPK4 knockdown glioma cells. The results showed that p‐AKT (T308) and p‐AKT (S473) levels decreased after MAPK4 silencing. To determine whether MAPK4 activates mTORC2 and the downstream P‐AKT (S473), we measured the expression of the downstream molecules p‐PKC and PKC. A decreased level of p‐PKC was found, supporting the conclusion that MAPK4 activates mTORC2 (Figure 8D,E).

We also performed IHC to measure the p‐AKT (T308) and p‐AKT (S473) levels in human glioma tissues and in glioma tissues from the mice in the MAPK4 knockdown group in the intracranial glioma model. In human glioma tissues, MAPK4 levels correlated significantly with the levels of p‐AKT (T308) and p‐AKT (S473) (Figure 8F). Moreover, the p‐AKT (S473) level was downregulated in glioma tissues from MAPK4 knockdown mice (Figure 8G). Our results suggest that MAPK4 may promote proliferation and migration via the AKT/mTOR pathway.

### 3.8 MAPK4 is associated with immune infiltration and with the expression of immune checkpoints

**FIGURE 9:**
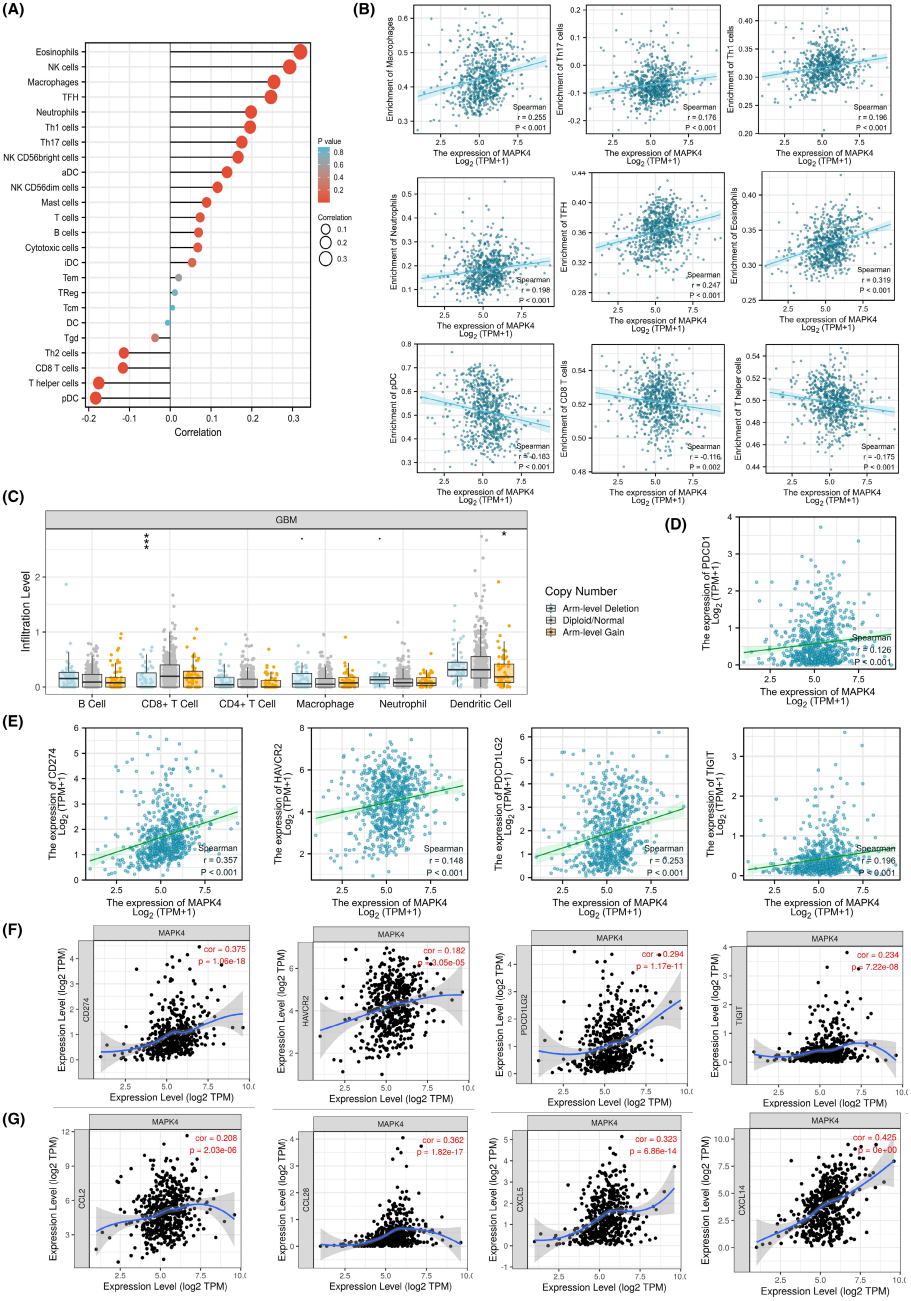
MAPK4 is associated with immune infiltration and with the expression of immune checkpoints. (A) Lollipop Chart showed the correlation between immune cell score and MAPK4 expression by ssGSEA algorithms in TCGA dataset. (B) The correlation between immune cell score and MAPK4 expression by ssGSEA arithmetic in TCGA dataset. (C) SCNAs module showed the comparison of tumor infiltration levels among tumors with different somatic copy number alterations for MAPK4. SCNAs are defined by GISTIC 2.0. Relations between the expression of MAPK4 and PD‐1 (D), PD‐L1, HAVCR2, PDCD1LG2 and TIGIT (E) in glioma in TCGA dataset. (F) Correlation of MAPK4 expression with PD‐L1, HAVCR2, PDCD1LG2 and TIGIT in GBM adjusted by tumor purity using TIMER.(G) Correlation of MAPK4 expression with CCL2, CCL28, CXCL5 and CXCL14 in GBM adjusted by tumor purity using TIMER. *p < 0.05, ***p < 0.001.

Considering that immune‐related signaling pathways were enriched in the GSEA, we analyzed the relationship between MAPK4 expression and immune infiltration in gliomas to explore the role of MAPK4 in the immune regulation of glioma. Notably, MAPK4 expression was positively correlated with tumor infiltration by macrophages, Th1 cells, Th17 cells, follicular helper T cells (TFHs), neutrophils and eosinophils but negatively correlated with infiltration by CD8^+^ T cells, plasmacytoid DCs (pDCs) and T helper cells (Figure 9A,B). We also performed an analysis of immune tumor infiltration levels among GBM with different somatic copy number alterations in MAPK4. Decreased infiltration of CD8^+^ T cells in gliomas with arm‐level deletion and decreased infiltration of dendritic cells in gliomas with arm‐level gain were found in GBM compared with gliomas with normal somatic copy numbers of the MAPK4 gene (Figure 9C). Thus, MAPK4 expression may be correlated with immune infiltration of the glioma microenvironment.

Programmed cell death protein 1 (PD‐1/PDCD1), programmed cell death 1 ligand 1 (PD‐L1/CD274), hepatitis A virus cellular receptor 2 (HAVCR2/TIM3), programmed cell death 1 ligand 2 (PDCD1LG2), and T‐cell immune‐receptor with Ig and ITIM domains (TIGIT) are vital immune checkpoint proteins that are promising cancer immunotherapy targets. As shown in Figure 9D–F, MAPK4 expression was significantly positively correlated with expression of the main immunoinhibitory checkpoint molecules PD‐1, PD‐L1, HAVCR2, PDCD1LG2 and TIGIT in glioma. Moreover, MAPK4 expression was significantly positively correlated with the expression of chemokines such as CCL2, CCL28, CXCL5 and CXCL14 (Figure 9G). These data suggest that MAPK4 may participate in immune infiltration and the expression of immune checkpoint proteins in the glioma microenvironment.

## 4 DISCUSSION

Our work demonstrates that MAPK4 is overexpressed in glioma and that it promotes the proliferation and migration of glioma cells via the AKT/mTOR pathway. Knockdown of MAPK4 suppressed proliferation and migration and induced cell cycle arrest in GBM cells. MAPK4 silencing markedly inhibited the growth of primary glioma. Our results are consistent with a recent report that presented evidence that MAPK4 is a novel therapeutic target in prostate cancer, especially castration‐resistant prostate cancer.^10^ Currently, treatment options for glioma patients are severely limited even though cancer therapies have been developed over the past decades, and new targets for therapeutic intervention are urgently needed.^16^, ^17^ Our study provides evidence that targeting MAPK4 may be a promising therapeutic avenue for GBM that shows significant expression of MAPK4.

Here, we have revealed that MAPK4 is significantly correlated with poor clinicopathological characteristics and disease progression in patients with glioma. Prognostic biomarkers play an important role in predicting clinical outcome and selecting appropriate therapy for patients in clinical practice.^18^, ^19^ Biomarkers were first used to classify gliomas by the World Health Organization in 2016 and received higher emphasis than ever before in 2021.^20^ Primary genetic markers such as IDH mutations, CDKN2A deletions, 1p/19q‐codeletions, H3F3A alterations, MGMT promoter methylation status and EGFR amplification are used in the glioma classification system.^20^, ^21^ To classify and grade gliomas and select appropriate therapy more precisely, more accurate and efficient diagnostic and prognostic biomarkers for glioma are needed. The usefulness of MAPK4 in the diagnosis and prognosis of glioma was analyzed using data from multiple public databases and confirmed in our glioma pathologic specimens. Nevertheless, there are limitations to finalize the MAPK4 as the novel diagnostic marker and prognostic indicator of glioma. We present evidence that MAPK4 may be an efficient diagnostic marker and prognostic indicator of glioma.

Functional state analyses performed at single‐cell resolution show that MAPK4 expression in glioma correlates with proliferation, metastasis, stemness, inflammation and angiogenesis. Our results confirm that MAPK4 promotes glioma progression by facilitating proliferation and migration both in vitro and in vivo, consistent with the previously reported roles of MAPK4 in breast cancer and prostate cancer.^10^, ^11^, ^12^ Signaling pathways such as the MAPK, PI3K, focal adhesion‐PI3K/AKT/mTOR, and EGFR pathways were enriched in gliomas with high MAPK4 expression. Considering that MAPK4 is an atypical member of the MAPK family, enrichment of the MAPK signaling pathway in the high MAPK4 expression group is reasonable. The AKT/mTOR pathway plays a crucial role in regulating cell survival, proliferation and metabolism. AKT (S473) is mainly activated by mTORC2.^11^ A previous study showed that MAPK4 activates AKT T308 by directly binding to AKT.^10^ mTORC1 is a vital hub that integrates extracellular and nutrient signals and thereby modulates cell growth, autophagy, and metabolism. Our data demonstrate that P‐AKT (T308) and P‐AKT (S473) levels are downregulated in glioma cells after MAPK4 knockdown, consistent with the positive correlation of P‐AKT (T308) and P‐AKT (S473) with MAPK4 expression in human glioma tissue. These results support the idea that MAPK4 facilitates the proliferation and migration of glioma cells through a mechanism that involves the activation of AKT. Aberrant EGFR signaling, which is also enriched in glioma with high MAPK4 expression according to GSEA, also promotes the proliferation, angiogenesis, invasion and metastasis of cancer by downstream RAS–RAF–MEK–ERK, PI3K/AKT and PLC gamma‐PKC.^22^, ^23^, ^24^ We also provide evidence that MAPK4 promotes the activation of PKC, a downstream effector of mTORC2, a finding that supports a pattern in which MAPK4 activates P‐AKT (S473).^10^ We speculate that the EGFR signaling pathway acts upstream of MAPK4 in glioma, and future work is aimed at determining whether MAPK4 is regulated by the EGFR signaling pathway.

Pathways such as neutrophil degeneration, cytokine and cytokine receptor interaction, the Toll‐like receptor signaling pathway, interferon signaling and interleukin 6 family signaling were enriched in the gliomas with high MAPK4 expression. MAPK4 expression correlates negatively with infiltration by CD8^+^ T cells, plasmacytoid DC cells and T helper cells and correlates positively with the expression of inhibitory immune checkpoint proteins, which have been recognized as immunotherapy targets in cancer.^25^, ^26^ The role of MAPK4 in immunity is largely unknown. Inhibition of MAPK4 ameliorated the activation of CD4+ T cells in mice with autoimmune hepatitis.^27^ Loss of MAPK4 inhibited proinflammatory cytokine production and altered the composition of immune cells, including neutrophils, macrophages and γδT^+^ cells, in an LPS‐induced murine ALI model.^28^ Our work suggests a probable role for MAPK4 in tumor immune regulation and facilitation of glioma progression. A limitation of our work is that the role of MAPK4 in immune regulation was explored only through bioinformatics analysis without experimental validation; thus, further investigation is needed. Nowadays, great progress in cancer immunotherapy has been witnessed. As the ability to penetrate the blood–brain barrier, immunotherapy is becoming a promising strategy for gliomas.^17^, ^29^ Our findings shed new light on the potential of MAPK4 as a possible target in the immune regulation of glioma and in immunotherapy.

## 5 CONCLUSION

Taken together, our findings demonstrate that MAPK4 plays a promoting role in glioma. MAPK4 functions as a prognostic indicator in glioma and promotes the proliferation and migration of glioma cells via the AKT/mTOR pathway. MAPK4 blockade might be a promising strategy for the discovery of plausible and attractive drug targets for glioma. Additionally, MAPK4 expression correlated negatively with the presence of antitumor immune cells and positively with the expression of immune‐inhibited checkpoint proteins in the glioma microenvironment yielding new insight into the promoting role of MAPK4. Further studies of the detailed mechanisms by which MAPK4 exerts its influence on the glioma immune microenvironment will have great significance.

## AUTHOR CONTRIBUTIONS

**Jing Ren:** Conceptualization (lead); data curation (lead); formal analysis (lead); funding acquisition (lead); project administration (equal); software (lead); writing – original draft (lead); writing – review and editing (lead). **Shijun Zheng:** Data curation (equal); project administration (equal); validation (equal); writing – review and editing (equal). **Lei Zhang:** Conceptualization (equal). **Jia Liu:** Conceptualization (equal); project administration (equal); resources (equal). **Haowei Cao:** Formal analysis (equal). **Sicheng Wu:** Software (equal). **Yixin Xu:** Conceptualization (equal); data curation (equal); visualization (equal); writing – original draft (equal). **Jinmin Sun:** Conceptualization (equal); data curation (lead); investigation (equal); methodology (lead); project administration (lead); resources (equal); software (equal); supervision (equal); validation (equal); visualization (equal); writing – original draft (equal); writing – review and editing (equal).

## FUNDING INFORMATION

This work is supported by the Natural Science Foundation of Jiangsu Province in China (BK20190984), National Natural Science Foundation of China (82002516), Natural Science Fund for Colleges and Universities in Jiangsu Province (19KJB310023) and Xuzhou Science and Technology Program (KC21170).

## CONFLICT OF INTEREST STATEMENT

The authors declare that they have no competing interests.

## ETHICS APPROVAL AND PATIENT CONSENT STATEMENT

The glioma specimens were collected with the informed consent of the patients, from the Affiliated Hospital of Xuzhou Medical University between 2016 and 2017. This study obtained the approval from Ethics Committee of the Affiliated Hospital of Xuzhou Medical University (Approval number. XYFY2018‐KL056‐01) and conducted in conformity to the Declaration of Helsinki. Bioinformatics data used in this article were from open assessing databases, and no permission was required.

## Data Availability

The datasets supporting the conclusions of this article are available in the TCGA repository (https://www.cancer.gov/about‐nci/organization/ccg/research/structural‐genomics/tcga), CGGA repository (http://www.cgga.org.cn/) and GEO repository (GSE131928, GSE102130, GSE57872, GSE103224, GSE102130, GSE57872 and GSE103224, https://www.ncbi.nlm.nih.gov/geo/). GTEx repository (https://www.genome.gov/Funded‐Programs‐Projects/Genotype‐Tissue‐Expression‐Project). Functional states of MAPK4 were analyzed by CancerSEA web server (http://biocc.hrbmu.edu.cn/CancerSEA/home.jsp). TIMER database analyses in present study are available in https://cistrome.shinyapps.io/timer/. LinkedOmics database analysis is available in http://www.linkedomics.org/login.php. STRING database analysis during the current study is available in https://cn.string‐db.org/. In addition, since pathological and clinical data involve patient privacy, if you want to obtain it or other information, please contact the corresponding author.
